# A Phase II Multi-institutional Clinical Trial Assessing Fractionated Simultaneous In-Field Boost Radiotherapy for Brain Oligometastases

**DOI:** 10.7759/cureus.6394

**Published:** 2019-12-16

**Authors:** George Rodrigues, Slav Yartsev, David Roberge, Robert MacRae, Wilson H Roa, Valérie Panet-Raymond, Giuseppina Laura Masucci, Brian P Yaremko, David D'Souza, David Palma, Tracy Sexton, Edward Yu, Jason Pantarotto, Belal Ahmad, Barbara Fisher, A. Rashid Dar, Carole Lambert, Gregory Pond, Keng Yeow Tay, Glenn Bauman

**Affiliations:** 1 Radiation Oncology, London Regional Cancer Program, London Health Sciences Centre, London, CAN; 2 Medical Physics, London Regional Cancer Program, London Health Sciences Centre, London, CAN; 3 Radiation Oncology, University of Montréal Health Centre, Montréal, CAN; 4 Radiation Oncology, Ottawa Hospital Research Institute, University of Ottawa, Ottawa, CAN; 5 Oncology, Cross Cancer Institute, University of Alberta, Edmonton, CAN; 6 Radiation Oncology, McGill University Health Centre, Montréal, CAN; 7 Radiation Oncology, Centre Hospitalier de l'Université de Montréal (CHUM), Montréal, CAN; 8 Radiation Oncology, Victoria Hospital, London Health Sciences Centre, London, CAN; 9 Oncology, Schulich School of Medicine and Dentistry, Western University, London, CAN; 10 Radiation Oncology, University of Ottawa, Ottawa, CAN; 11 Radiation Oncology, University of Miami Miller School of Medicine, Miami, USA; 12 Radiation Oncology, Schulich School of Medicine & Dentistry, Western University, London, CAN; 13 Radiation Oncology, Centre hospitalier de l'université de Montréal (CHUM) - Hôpital Notre-Dame, Montréal, CAN; 14 Epidemiology and Public Health, Juravinski Cancer Centre-McMaster University, Hamilton, CAN; 15 Radiology, London Health Sciences Centre, London, CAN

**Keywords:** radiotherapy, brain metastases, oligometastases, clinical trial, phase ii

## Abstract

Purpose/Objective

Published preclinical and phase I clinical trial data suggest that fractionated lesional radiotherapy with 60 Gy in 10 fractions can serve as an alternative approach to single fraction radiosurgical boost for brain oligometastases.

Methods and Materials

A phase II clinical trial (NCT01543542) of a total of 60 Gy in 10 fractions of lesional (one to three) radiotherapy (given simultaneously with whole-brain helical tomotherapy with 30 Gy in 10 fractions) was conducted at five institutions. We hypothesized that fractionated radiotherapy would be considered unsuitable if the median overall survival (OS) was degraded by two months or if six-month intracranial control (ICC) and intracranial lesion (ILC) were inferior by 10% compared with the published RTOG 9508 results.

Results

A total of 87 patients were enrolled over a 4.5-year accrual period. Radiological lesion and extralesional central nervous system progression were documented in 15/87 (17%) and 11/87 (13%) patients, respectively. Median OS for all patients was 5.4 months. Six-month actuarial estimates of ICC and ILC were 78% and 89%, respectively. However, only the ILC estimate achieved statistical significance (p=0.02), demonstrating non-inferiority to the a priori historical controls (OS: p=0.09, ICC=0.31). Two patients developed suspected asymptomatic radionecrosis.

Conclusions

The phase II estimates of ILC were demonstrated to be non-inferior to the results of the RTOG 9508.

## Introduction

The radiotherapeutic treatment of brain metastases has shifted considerably in the last two decades in response to clinical trial evidence [1-2]. Traditionally, treatment of brain metastases was often limited to palliative whole-brain radiotherapy (WBRT); however, clinical trial investigations of treatment intensification of index brain lesions with surgery and radiosurgery have demonstrated improvements in various clinical endpoints such as overall survival (OS), intracranial cancer control, and steroid dependency [3-7]. The additive role of WBRT in the context of surgery [8-9] and radiosurgery has also been extensively investigated in the medical literature leading to a Choosing Wisely statement against the routine use of adjuvant WBRT after single-fraction stereotactic radiosurgery (SRS) [9-15]. 

The combination of image-guided procedures and advanced, highly conformal treatment planning paradigms has allowed for the development of non-invasive fractionated alternatives to SRS [16-17]. This fractionated alternative has been reviewed and it suggested that fractionated SRT (fSRT) may be associated with “clinical benefits including overall survival, local control and acceptable toxicity” [18]. This review also illustrated potential associations between lesional dose and local control, suggesting that fractionated treatment can be considered as a reasonable alternative “as long as the equivalent dose of radiotherapy delivered to the lesion(s) is sufficiently intense” [18]. Although there is optimism that fSRT may prove to be beneficial, the clinical benefits have not been firmly established and require further evaluation in well-conducted prospective clinical trials.

The role of fSRT was previously investigated in the context of a preclinical study and phase I clinical trial [16, 19]. In the preclinical study, the feasibility of treatment planning for lesional dose escalation to 60 Gy in 10 fractions while respecting organ at risk dose tolerances was determined. These treatment plans were verified in phantoms and demonstrated improved organ-at-risk sparing relative to comparable plans combining WBRT with single-fraction radiosurgery boost [16]. As a result of this preclinical work, a phase I clinical trial was initiated to investigate the safety of a series of escalating lesional doses (35, 45, 50, 55, and 60 Gy) in the context of simultaneous in-field boost (SIB) technique with a WBRT dose of 30 Gy in 10 fractions [19] for patients with one to three brain metastases. This clinical trial demonstrated that 60 Gy in 10 fractions was feasible and safe and that this fractionation schedule was selected for further phase II testing [20].

The purpose of this study was to determine and report on important clinical endpoints (OS, lesional control, and intracranial control [ICC]) related to the fSRT/SIB treatment of oligometastatic brain cancer. The results of this trial are placed within the historical control context of the pivotal RTOG 9508 clinical trial demonstrating superiority of SRS plus WBRT versus WBRT alone [7] as well as the changing practice of the use of integrated WBRT for oligometastatic brain cancer.

## Materials and methods

This phase II clinical trial was approved by the institutional research ethics board (UWO REB 16776) and registered (clinicaltrials.gov NCT01543542) prior to trial initiation. Full protocol details are available in the peer-reviewed study protocol publication [20]; however, essential details are presented below.

Study Objective

The primary objective of this clinical trial is to assess the median OS, intralesional control ([ILC] local control of index lesions treated to 60 Gy on protocol) at six months, and ICC (both lesional control and extralesional control) at six months for a course of fSRT given at a total dose of 60 Gy in 10 fractions for one to three brain metastases (delivered simultaneously with 30 Gy in 10 fractions WBRT). The inclusion of WBRT was considered to be a routine treatment during the initiation and conduct of this clinical trial. The null hypothesis for the study was that fSRT/SIB is not inferior to the RTOG 9508 outcomes for all three of these outcomes. The study’s alternative hypothesis was that fSRT/SIB treatment is inferior to any reported historical RTOG 9508 outcomes of OS, ILC, and ICC related to SRS treatment.

Study Design Overview

This study was designed as a single arm multi-institutional Canadian (London Regional Cancer Program, London, Ontario; Ottawa Integrative Cancer Centre, Ottawa, Ontario; CHUM - Centre hospitalier de l'Université de Montréal, Montreal, Quebec; McGill University, Montreal, Quebec; and Cross Cancer Institute, Edmonton, Alberta) clinical trial without blinding or randomization. Patients were accrued into various study bins to ensure the proportion of patients with a lung primary, and one vs. two to three metastases would be similar to that reported in the RTOG 9508 trial [20]. The fSRT/SIB platform selected for the trial was helical tomotherapy as this image-guided, intensity-modulated radiation delivery system was available at all the participating sites. Planning procedures for protocol treatment on the helical tomotherapy (TomoTherapy, Accuray Inc., Morges, Switzerland) have been comprehensively discussed in previous publications [16, 19-20].

Inclusion criteria for this phase II clinical trial included the following: a histological diagnosis of primary cancer, a contrast-enhanced MRI demonstrating one to three metastases (all less than or equal to 3 cm in maximum diameter) within six weeks of study enrolment, patient age greater than 18 years, Karnosky Performance Status (KPS) greater than or equal to 70, available for subsequent follow-up appointments and diagnostic testing, and an anticipated survival of more than three months. Patients with extracranial disease were allowed on protocol as long as that disease was either previously controlled with anticancer therapy (e.g., surgery, chemotherapy) or will be treated after protocol radiotherapy has been completed.

Exclusion criteria included any metastases within 5 mm of the brainstem or optic apparatus, cytological or imaging evidence of leptomeningeal spread, intracranial extension of an osseous metastasis or evidence of intraventricular or subependymal growth, prior cranial radiotherapy, concurrent cytotoxic chemotherapy during radiation therapy, and contraindications to MRI or gadolinium contrast.

Study Endpoints and Sample Size

Patients treated with SRS/WBRT on the SRS arm of the RTOG 9508 trial were reported to have a median OS of 6.5 months, six-month actuarial ICC of 85%, and six-month actuarial ILC of 90%. We hypothesized that protocol fSRT would be considered unacceptable if the median OS was reduced by two months or if six-month ICC or ILC were inferior by 10% compared with the RTOG 9508 results. Therefore, if our phase II trial demonstrated any of the following, protocol fSRT/SIB treatment would be considered to be inferior: OS ≤ 4.5 months, six-month ICC rate ≤ 75%, or six-month ILC rate ≤ 80%. To account for multiple testing, statistical assessment of each study outcome was assessed at the α = 0.025 and β = 0.10 level. Therefore, for OS, a total of 87 patients were required. Fewer patients (67 and 50 patients) were required for the six-month ICC and ILC endpoints; therefore, total accrual was set at 87 patients to assess all three outcomes of interest (OS, ICC, ILC). The initial sample size for the study was calculated to be 93 patients [20]; however, due to an extended accrual period (4.5 years vs. 3 years), a final sample size of 87 patients was calculated to be sufficient for completion of the clinical trial and was approved by the trial data safety monitoring committee.

Study Procedures

All patients underwent contrast-enhanced brain MRI within six weeks of study enrolment, a history including steroid and anti-convulsant usage, a physical examination, a screening neurological examination with the Mini-Mental Status Examination (MMSE), Functional Assessment of Cancer Therapy-Brain (FACT-Br) health-related quality of life (HRQoL) questionnaire completed during the enrolment visit, and a baseline National Cancer Institute Common Toxicity Criteria Version 3 (NCI CTC V3) toxicity assessment. Follow-up visits and assessments (KPS, NCI CTC V3 toxicity, FACT-Br HRQoL, steroid, and anticonvulsant medications) were at six weeks as well as at 3, 6, 9, 12, 18, and 24 months following protocol treatment. Protocol mandated contrast-enhanced MRI brain scans occurred at three and six months after radiation therapy completion. Other CT/MRI-based imaging occurred thereafter according to institutional guidelines. Overall radiological follow-up compliance in this study was 98% (151/154 expected scans).

Statistical Analysis Plan

Descriptive statistics were used to summarize the patient population, including tumor- and treatment-related parameters, treatment toxicities, causes of death, location of intracranial, intralesional and extracranial progression, and performance status. Time-to-event outcomes (OS, ICC, and ILC) were estimated using the Kaplan-Meier method in order to report on all three primary endpoints of the study (median OS, six-month ICC, and six-month ILC). One-sided t-tests were used to determine the non-inferiority of the point estimates compared with the a priori defined RTOG historical controls. Patients were censored at the time of the last radiological study to calculate lesional control and ICC. Secondary endpoints such as health-related quality of life and MMSE will be reported after completion of two years of follow-up on all study subjects.

## Results

Patient Population

A total of 87 patients were enrolled on the clinical trial over a 4.5-year accrual period from five institutions (London, ON [n=49]; Montreal-CHUM, QC [n=26], Ottawa, ON [n=6], Edmonton, AB [n=4], and Montreal-McGill, QC [n=2]). The mean age of participants was 61 years (range: 23-84 years), and 54/87 (62%) were females. Median KPS and MMSE were 80/100 (range: 70-100) and 29/30 (range: 21-30), respectively. In terms of primary histology, 54/87 (62%) had lung cancer, 10/87 (11%) had breast cancer, 9/87 (10%) had gastrointestinal, 5/87 (6%) had kidney, and 9/87 (10%) had other assorted primaries and histologies. Primary disease was treated and controlled in 57/87 (65%), with 60/87 (69%) with known extracranial disease. A total of 49/87 (56%) of patients had prior chemotherapy and 42/87 (48%) had prior or concurrent extracranial radiotherapy. There were 42/87 (48%) with a single brain metastases, 29/87 (33%) with two metastases, and 16/87 (18%) with three metastases. Median lesion size (n=148) was 13 mm (range: 2-30 mm). A comparison of various baseline factors versus those related specifically to the SRS plus WBRT arm of RTOG 9508 is summarized in Table 2.

**Table 1:** Patient demographics of phase II clinical trial patients versus RTOG 9508 RTOG, Radiation Therapy Oncology Group; SRS, stereotactic radiosurgery; WBRT, whole-brain radiotherapy; KPS, Karnosky Performance Status

Variable	Phase II Clinical Trial	RTOG 9508 SRS+WBRT
Age	Mean: 61 years	Mean: 59 years
KPS	Median: 80	Median: 90
Primary lung	54/87 (62%)	105/164 (64%)
Number of brain metastases		
1	42/87 (48%)	92/164 (56%)
2	29/87 (33%)	39/164 (24%)
3	16/87 (18%)	33/164 (20%)
Primary controlled/absent	57/87 (65%)	126/164 (77%)
Extracranial disease	60/87 (69%)	112/164 (68%)

**Table 2:** Summary of radiation treatment PTV, planning target volume; NR, not reported

Structure	D_99%_ Median (Range)	D_95%_ Median (Range)	D_1%_ Median (Range)
Whole-brain PTV	29.5 Gy (27.10-30.7)	30.0 Gy (29-30.8)	53.7 Gy (30.6-61.1)
Lesional PTV	59.8 Gy (45.1-61.4)	60.1 Gy (48.9-62.0)	62 Gy (53.4-65.4)
Lens	3.2 Gy (2.1-7.1)	NR	4.2 Gy (2.9-9.5)
Brainstem	28.5 Gy (1.7-30.7)	NR	32.0 Gy (29.4-38.8)

Treatment Toxicity 

Observed acute toxicities potentially related to protocol treatment included one episode of grade four seizures in one patient. Ten other grade 3 acute events were observed during the conduct of the trial including six patients with worsening energy level/weakness, one patient with seizures, one patient with confusion, one patient with tumor flare, and one patient with headaches. Commonly documented grade 1 and 2 toxicities included reduction in energy level in 31/87 (36%), alopecia in 28/87 (32%), headache in 11/87 (13%), and nausea/vomiting in 9/87 (10%). Two (2%) patients developed suspected radionecrosis of their index lesions on follow-up MRI. Neither had tissue confirmation, as they were asymptomatic, and the radionecrosis findings remained stable over time.

Primary Outcome Analyses

**Figure 1:**
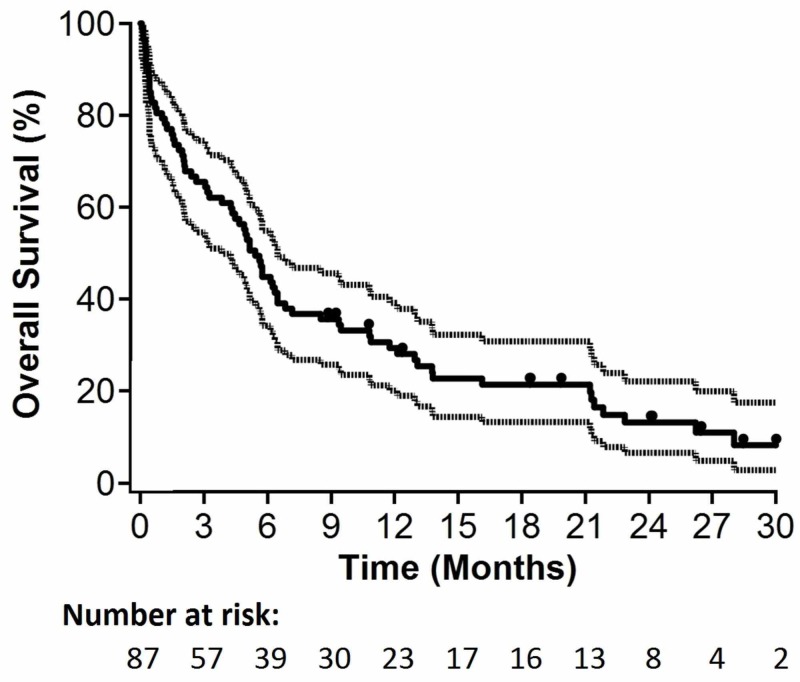
Kaplan-Meier plots for overall survival (n=87).

**Figure 2:**
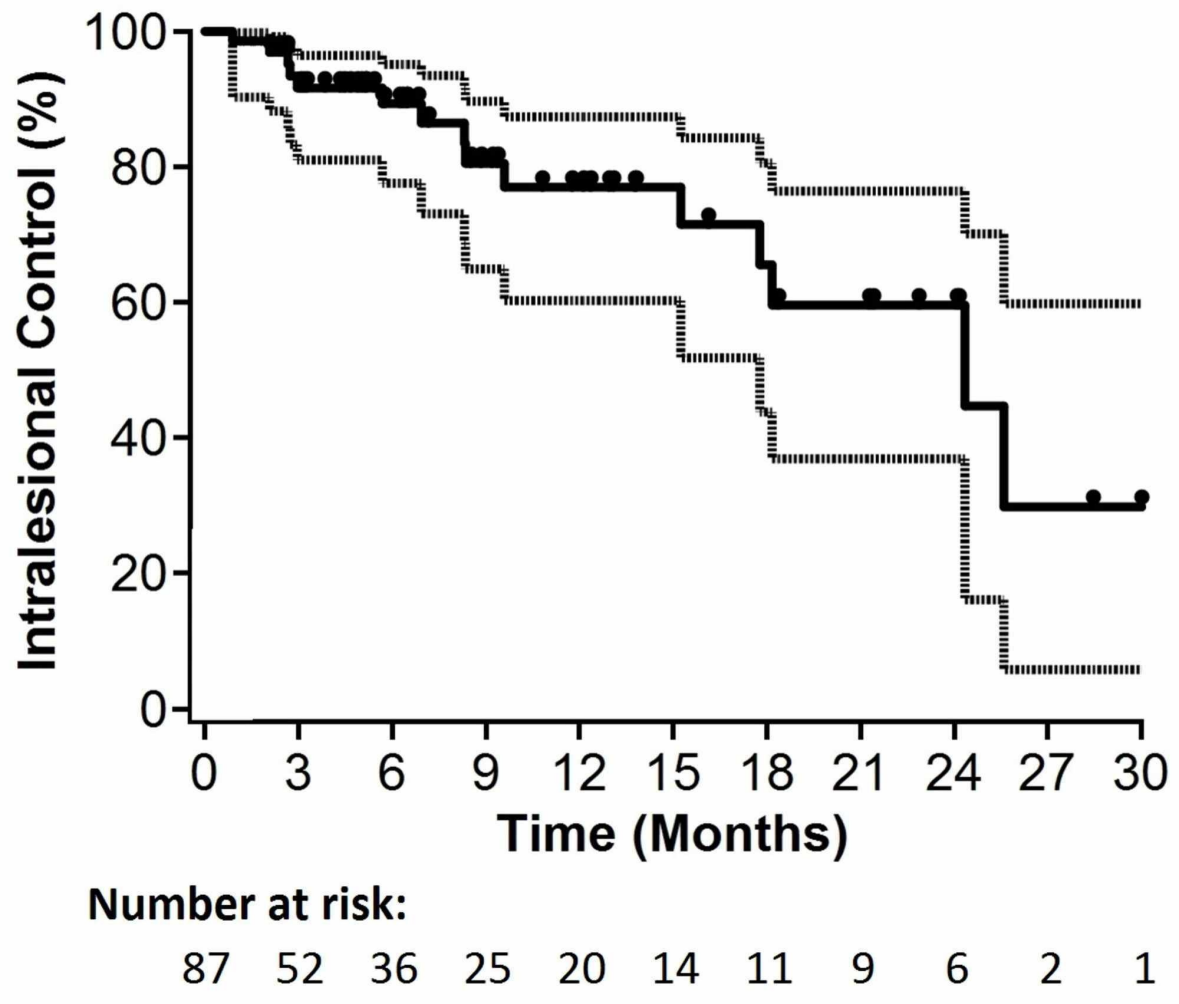
Kaplan-Meier plot for intralesional control (n=87).

**Figure 3:**
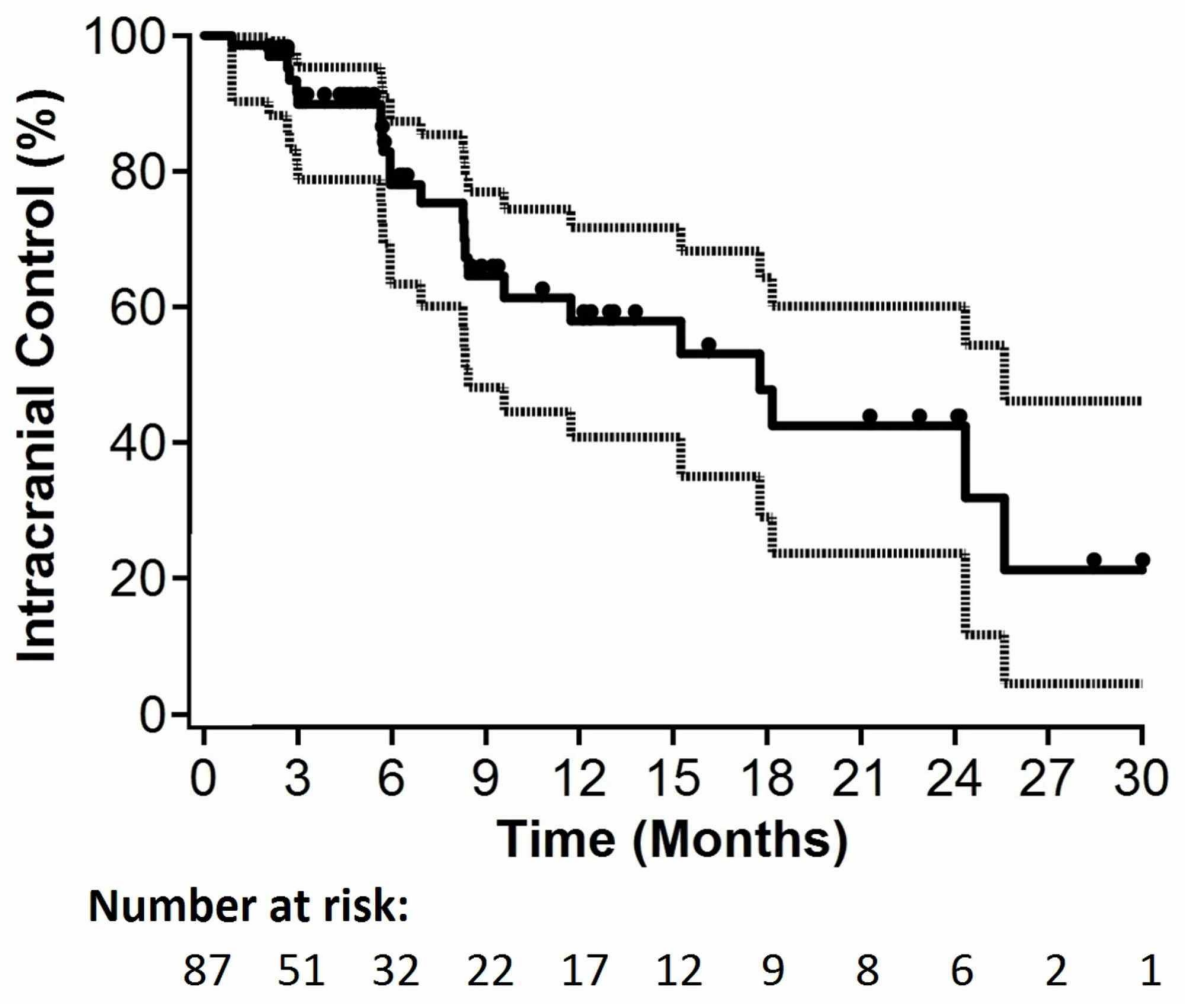
Kaplan-Meier plot for intracranial control (n=87).

Radiological lesion and extralesional central nervous system progression were documented in 15/87 (17%) and 11/87 (13%) patients, respectively. Median OS for all patients was 5.4 months (95% confidence interval [CI]: 3.8-6.5 months; p=0.09 for non-inferiority). Median time to lesional progression and median time to intracranial progression were 5.0 and 4.9 months, respectively. Six-month actuarial estimates of ICC and ILC were 78% (95% CI: 63-87%; p=0.31 for non-inferiority) and 89% (95% CI: 78-95%; p=0.02 for non-inferiority), respectively. Kaplan-Meier curves for OS, ILC, and ICC are depicted in Figures 1-3, respectively.

## Discussion

This multi-institutional prospective phase II trial suggests that this fSRT/SIB approach is both safe and efficacious for the treatment of oligometastatic brain disease when compared to important clinical endpoints observed in the SRS and WBRT arm of the pivotal RTOG 9508 phase III clinical trial. OS, ILC, and ICC associated with the protocol fSRT/SIB treatment were within the a priori defined acceptability criteria that underpinned the sample size calculation. Potential residual variations in baseline covariate characteristics (such as the type of primary cancer, number of brain metastases, presence of extracranial disease, and performance status; see Table 1) between this study population and RTOG 9508 study population are likely to partly explain the small differences in the point estimates of OS and ICC (as well as not being able to demonstrate statistical non-inferiority); however, the six-month ILC estimate of 89% for fSRT/SIB compares favorably (and was statistically non-inferior) with the 90% estimate reported in the SRS and WBRT arm of the RTOG 9508 study.

Given the current lack of a randomized phase III trial evaluating this form of treatment versus SRS, it is unlikely that this trial will shift clinical practice in centers with SRS clinical programs. However, from a broader perspective, SRS programs do not exist at all radiation treatment centers due to the specialized nature of this treatment requiring significant programmatic resources [21-22]. This clinical trial has demonstrated that an image-guided fSRT approach using suitable immobilization and highly conformal treatment delivery is associated with clinical outcomes comparable to those achieved with traditional radiosurgery approaches. Our findings suggest that the treatment as delivered in this study could be an alternative approach for appropriate patients who do not or cannot travel to specialized SRS centers for radiosurgery treatment.

Other investigators have described results associated with similar fSRT (including simultaneous WBRT) treatment using volumetric modulated radiation therapy (VMAT) in a five-fraction treatment schedule. Nichol et al have described in abstract form their prospective phase II trial of 50 Gy in five fractions (lesion dose) given simultaneously with 20 Gy in five fractions of WBRT in 60 patients with 1-10 brain metastases [23]. Although they were able to demonstrate three-month outcomes similar to the SRS and WBRT arm of RTOG 9508, they also observed a 7% risk of symptomatic grade 3 or 4 radionecrosis associated with their fSRT/SIB approach. Lagerwaard et al. published both their preclinical [24] and clinical experience [25-26] with a similar VMAT with simultaneous integrated boost approach using a total lesional dose of 40 Gy in five fractions with 20 Gy in 5 fractions WBRT for one to six brain metastases [24-26]. In their latest update of 65 patients in abstract form [26], they reported a favorable median OS of 9.6 months; however, the overall radionecrosis rate was 11% (5% symptomatic).

All three studies of fSRT/SIB demonstrate that clinical outcomes comparable with the SRS and WBRT arm of the RTOG 9508 study are achievable with either 5- or 10-fraction fSRT/SIB. There are several reasons why there may be a difference in observed radionecrosis rates between the 5-fraction studies and the current 10-fraction approach. Firstly, the other two trials allowed for more than three lesions (Nichol), and some of the differences in reported radionecrosis rates may be simply due to the higher chance of this event occurring when additional lesions are treated. Secondly, the fractionation schedules are different, with the five-fraction technique using a daily fraction dose of 8-10 Gy as opposed to 6 Gy in our approach. Thirdly, margin selection around the index lesions may be important. Both of the five-fraction approaches used additional margins around the lesional GTV, whereas our approach did not use additional margin, allowing both the lesional dose fall-off and the WBRT component to cover the peri-lesional area. Additionally, the median survival reported in these two abstracts is longer than in our experience, and, therefore, these patients may be at a more baseline risk of developing radionecrosis during their follow-up time.

The integration of WBRT with SRS has been an area of controversy as exemplified by various clinical studies, review articles, randomized controlled trials, and two meta-analyses [9-14, 27-29]. The inclusion of WBRT has been shown to improve distant brain control and potentially lesion control; however, no OS benefit has been demonstrated by the inclusion of this modality. Furthermore, inclusion of WBRT is associated with negative neurocognitive effects and potential reduction in OS in patients aged 50 years or less [14]. This collective medical literature has led the American Society of Radiation Oncology to publish the following Choosing Wisely statement: “Don’t routinely add adjuvant whole brain radiation therapy to stereotactic radiosurgery for limited brain metastases” [15].

How should then clinicians interpret and potentially apply our trial (initiated years prior to the Choosing Wisely statement) results given this movement away from the integration of WBRT in patients with oligometastatic brain disease? Some patients may still elect to receive WBRT as a component of their treatment to potentially avoid distant brain relapse and/or the frequent MRI surveillance scans that are required as part of the lesion alone strategy [30]. Clinical nomograms are available to predict the risk of distant brain failure to assist clinicians and patients in this decision. Another approach is to simply exclude the WBRT from the fSRT/SIB treatment, which would simplify the treatment planning process and reduce both whole-brain and hippocampal dose, thus improving the potential neurocognitive profile of this treatment. However, the outcomes associated with such a treatment have not been formally investigated in the context of a clinical trial.

The main limitation of this phase II historical controlled study is the absence of an SRS-based control arm to more precisely compare the important clinical outcomes described in this study. This trial attempted to control for two important confounders (lung primary and number of brain metastases; see Table 1); however, other known (performance status, extracranial disease; see Table 1) and unknown confounders prevent a completely unbiased comparison of results. Ideally, a phase III trial comparing this fSRT/SIB approach with SRS (either without WBRT or stratifying for WBRT use) would be able to show either equivalence or non-inferiority of the fSRT/SIB approach to the SRS approach. Such a trial would only be able to be conducted at cancer centers with both SRS and fSRT/SIB capability, potentially limiting the accrual (and feasibility) of a large phase III trial.

## Conclusions

Phase II estimates of all three endpoints studied in this clinical trial of fSRT/SIB for patients with one to three brain metastases compare favorably to the estimates of these endpoints observed in RTOG 9508. We recommend that fSRT to 60 Gy in 10 fractions can be considered as one of several alternative hypofractionated radiotherapy options to single-fraction SRS for the treatment of oligometastatic brain lesions. This treatment option is particularly relevant in practice locations without SRT apparatus and can be potentially adapted to deliver this treatment without the whole brain component or with hippocampal sparing.
